# *Ex Vivo* Pulmonary Oedema after *In Vivo* Blast-Induced Rat Lung Injury: Time Dependency, Blast Intensity and Beta-2 Adrenergic Receptor Role

**DOI:** 10.3390/biomedicines10112930

**Published:** 2022-11-15

**Authors:** Hanno Huwer, Yalda Hadizamani, Ueli Moehrlen, Uz Stammberger, Florian Gebhard, Lia Bally, Albrecht Wendel, Ulrich C. Liener, Rudolf Lucas, Jürg Hamacher

**Affiliations:** 1 Department of Cardiothoracic Surgery, Völklingen Heart Centre, 66333 Völklingen, Germany; 2 Pneumology, Clinic for General Internal Medicine, Lindenhofspital Bern, 3012 Bern, Switzerland; 3 Lungen-und Atmungsstiftung, Bern, 3012 Bern, Switzerland; 4 Department of Pediatric Surgery, University Children’s Hospital Zurich, 8032 Zurich, Switzerland; 5 STM ClinMedRes Consulting, 4056 Basel, Switzerland; 6 Department of Traumatology, Hand-, Plastic-, and Reconstructive Surgery, Center of Surgery, University of Ulm, Albert-Einstein-Allee 23, 89081 Ulm, Germany; 7 Department of Diabetes, Endocrinology, Clinical Nutrition and Metabolism Inselspital, Bern University Hospital, University of Bern, 3010 Bern, Switzerland; 8 Biochemical Pharmacology, Department of Biology, University of Konstanz, 78457 Konstanz, Germany; 9 Department of Trauma, Hand, Plastic and Reconstructive Surgery, University of Ulm-Surgical Center, Steinhövelstraße 9, 89075 Ulm, Germany; 10 Vascular Biology Center, Augusta University, Augusta, GA 30912, USA; 11 Department of Pharmacology and Toxicology, Augusta University, Augusta, GA 30912, USA; 12 Department of Medicine, Medical College of Georgia, Augusta University, Augusta, GA 30912, USA; 13 Medical Clinic V—Pneumology, Allergology, Intensive Care Medicine, and Environmental Medicine, Faculty of Medicine, Saarland University, University Medical Centre of the Saarland, D-66421 Homburg, Germany; 14 Institute for Clinical and Experimental Surgery, Faculty of Medicine, Saarland University, D-66421 Homburg, Germany

**Keywords:** blast lung injury, β_2_-adrenoceptor agonist, amiloride, terbutaline, formoterol, pulmonary oedema, inflammation, sodium transport

## Abstract

**Objective**: Current treatments for blast-induced lung injury are limited to supportive procedures including mechanical ventilation. The study aimed to investigate the role of post-trauma-induced oedema generation in the function of time and trauma intensity and the probable role of beta 2-adrenergic receptors (β_2_-ARs) agonists on pulmonary oedema. The study is conducted using an *ex vivo* model after an experimental *in vivo* blast-induced thorax trauma in rats. **Methods**: Rats were randomised and divided into two groups, blast and sham. The blast group were anaesthetised and exposed to the blast wave (3.16 ± 0.43 bar) at a distance of 3.5 cm from the thorax level. The rats were sacrificed 10 min after the blast, the lungs explanted and treated with terbutaline, formoterol, propranolol or amiloride to assess the involvement of sodium transport. Other groups of rats were exposed to distances of 5 and 7 cm from the thorax to reduce the intensity of the injury. Further, one group of rats was studied after 180 min and one after 360 min after a 3.5 cm blast injury. Sham controls were exposed to identical procedures except for receiving blast overpressure. **Results**: Lung injury and oedema generation depended on time after injury and injury intensity. Perfusion with amiloride resulted in a further increase in oedema formation as indicated by weight gain (*p* < 0.001), diminished tidal volume (Tv) (*p* < 0.001), and increased airway resistance (*p* < 0.001). Formoterol caused a significant increase in the Tv (*p* < 0.001) and a significant decrease in the airway resistance (*p* < 0.01), while the lung weight was not influenced. Trauma-related oedema was significantly reduced by terbutaline in terms of lung weight gain (*p* < 0.01), Tv (*p* < 0.001), and airway resistance (*p* < 0.01) compared to control blast-injured lungs. Terbutaline-induced effects were completely blocked by the β-receptor antagonist propranolol (*p* < 0.05). Similarly, amiloride, which was added to terbutaline perfusion, reversed terbutaline-induced weight gain reduction (*p* < 0.05). **Conclusions**: β_2_-adrenoceptor stimulation had a beneficial impact by amiloride-dependent sodium and therefore, fluid transport mechanisms on the short-term *ex vivo* oedema generation in a trauma-induced *in vivo* lung injury of rats.

## 1. Introduction

Blast injury occurs when an explosive system detonates and generates short-term, high-intensity blast waves with positive pressure followed by negative pressure, potentially pursued by blast wind of explosive gases [1]. Blast injuries are classified into various categories [2]. Primary blast injury occurs in a high-energetic explosion when an explosive shock wave causes barotrauma and passes through susceptible areas including the lungs, gastrointestinal tract, trachea, larynx, middle ear, and central nervous system [3,4]. In case of debris penetration into the body surface, secondary blast injury occurs and if blast waves relocate the victim or cause a building to collapse, tertiary blast injuries including penetrating or blunt force trauma takes place. Quaternary blast injury may arise when an explosion leads to injury by fire, radiation, gas or smoke and toxins [2,5]. Primary blast lung injury, either in the penetrating state or in the form of blunt lung injury, chiefly leads to pulmonary barotrauma or contusion. It may be combined with pneumothorax or pneumatocele, subcutaneous emphysema, tracheal injury, or pneumomediastinum [3,5,6]. Based on the nature and severity of the trauma, blast lung injury can cause transient discomfort to complex, multidimensional injuries and even sudden death [6,7,8,9].

Biologic consequences of blast lung injury may occur with latency, as in the initial phase after trauma the clinical signs of pulmonary dysfunction can be missing [10]. Blast lung injury can lead to respiratory failure. This is the consequence of a rupture of the alveolar capillary’s barrier and subsequent intrapulmonary haemorrhage, pulmonary oedema that may often lead to hypoxia, part of it further aggravated by severe ventilation–perfusion mismatch, but also by air embolism, and by pulmonary arterial bone marrow and fat embolism, and by the inflammatory and repair response as a specific sort of acute lung injury [11,12,13,14].

So far treatment has been chiefly limited to supportive care [8], mechanical ventilation and possibly corticosteroid therapy, evacuation of pneumothorax or effusions, and sometimes intensive care management [6,7,9,10,15].

β_2_-ARs are transmembrane proteins expressed in a variety of cells such as smooth muscle cells, immune cells, epithelial cells, skeletal muscle cells, and glandular cells [16]. β_2_-AR expression in the lung and in particular in alveoli has been found to be among the highest of all tissues [16]. Stimulation of β_2_-ARs launches several signalling mechanisms/pathways, which are important in physiological and pathophysiological aspects of cell life [16]. Indeed, they have been shown to exert a protective effect by enhancing alveolar and liquid clearance, therefore reducing pulmonary oedema [17].

The aim of the study was to investigate the modulation of the alveolar–capillary injury or permeability oedema evolution by time, by blast intensity, and by the beta receptor modulating drugs exploiting in vivo blast-induced lung injury in rats. We used a reliable, reproducible and predictive model of in vivo blast lung injury of rats followed by an *ex vivo* ventilated rat lung perfusion that enabled us to investigate pathophysiologic aspects of oedema following a blunt blast-induced thorax trauma. The combination of a relevant reproducible in vivo acute lung injury and the pathophysiological *ex vivo* assessment of oedema that allows pharmacological interventions with oedema-modulating substances has, to our knowledge, not been studied so far. Since amiloride as a sodium transport inhibitor in a species-dependent manner, inhibited 40–70% of basal alveolar fluid clearance [18], we used amiloride [19] to examine the role of sodium channels after trauma. We used the β_2_-adrenoceptor agonist’s terbutaline as the standard substance for oedema clearance and the clinically more important formoterol in low drug concentrations [20,21,22] to examine the role of β_2_-adrenoceptor, and the non-selective β-adrenergic receptor antagonist propranolol [19] to assess the impact of the substances on oedema evolution including airway function.

## 2. Methods

### 2.1. In Vivo Procedure

Details of *in vivo* procedures have been described in our previous study titled Characterization of pulmonary inflammatory response and repair after blast lung injury in rats by Hamacher et al. (submitted in 2022). Briefly, the rats were anaesthetised (Flow: 1.5 L/min oxygen, 4% Halothane^®^) and fixed in a supine position and received the analgesic (0.03 mg/kg BW Buprenorphine) subcutaneously. Next, they received a single blast wave. It was shown that pressure characteristics and lung damage were significantly related to the distance to the blast source. We generated a blast-induced blunt thorax trauma from a blast generator at their top of expiration at either 3.5, 5 or 7 cm distance from their sternum. Immediately after blast injury, rats received O_2_ (flow: 4–5 L/min) to get out of anaesthesia. To terminate the *in vivo* procedure rats were anaesthetised by intraperitoneal injection of pentobarbital sodium (160 mg/kg BW) at different time points after the blast including 4 min and 150 min after receiving blast wave. According to the ethical impact of the *in vivo* experiments and high statistical power of many read-outs, we followed the ethical recommendation to reduce the number of animals where possible, therefore limiting where possible the number of experiments. Of note, information for commercial material has been provided in supplementary materials.

Thereupon the study continues by *ex vivo* course of actions to evaluate the efficacy of medications in disorders caused by such blast injuries. Table S1 summarises the product information of used materials.

#### 2.1.1. Pressure Wave Monitoring

Different sensors at varying positions offered monitoring of the exposed blast pressure wave (Erne B., Abteilung Elektronik, Wissenschaftliche Werkstätten, Universität Konstanz):

1. A pressure transducer (PR-6ST-80400.XX-20, sensitivity: 39.7 mV/bar, excitation: 4 mA, natural frequency: <30 kHz; Keller AG, Winterthur, Switzerland) in the pressure reservoir determined the cracking pressure that corresponds to the rupture of the polyester film. The measured time course of the pressure wave curve indicated the velocity of the pressure increase followed by the decay.
2. Two pressure transducers (2 MI PAA 110-050-020, sensitivity: 8.03/7.99 mV/bar, excitation: 1 mA, natural frequency: >400 kHz; Keller AG, Winterthur, Switzerland) on both sides of the rat measured the pressure peaks on rat thorax level.
3. Another transducer (Halleffekt-IC 634-SS2, sensitivity: 7.5–10.6 mV/mT; RS Components GmbH, Mörfelden-Walldorf, Germany) recorded the breathing frequency of the animal. Therefore, a string connected with the breathing sensor was placed above the thoracic cage. Via this transducer, the valve was triggered automatically depending on the breathing situation (e.g., inspiration, expiration or resting expiratory position). In addition, the breathing frequency of the anaesthetised animals was recorded before and after the pressure wave exposure.

The measured pressure wave signals were transmitted through an amplifier (Burr-Brown, Texas Instruments GmbH, Freising, Germany) either to the computer (cracking pressure transducer) via an A/D converter (MAX 186, Maxim Corporate Headquarters, Sunnyvale, CA, USA) or (pressure peak transducer) to a HAMEG oscilloscope (HM 407, HAMEG instruments, VELMA, Großkrotzenburg, Germany). Pressure wave data were calculated by a developed software (Heine G., Abteilung Elektronik, Wissenschaftliche Werkstätten, Universität Konstanz) to determine the cracking pressure of the polyester film (Rp), wave form, pressure peaks (Pp(r), Pp(l)), impulse or area under the curve (AUC(r), AUC(l)), duration (t(r), t(l)) and pre- and post-breathing frequency (Fpre, Fpost). For the calculation of these parameters, the following equations were used:

Pressure peaks (Pp(r), Pp(l)):

$$\bar{P}=\frac{\sum_{i=m1}^{i=m}U_{i}}{t_{2}-t_{1}}$$

(U = voltage; *t* = time), whereas the voltage U is proportional to the pressure *P* (cracking pressure transducer: 0.5 bar = 1 bar, pressure peak transducer: 0.1 bar = 1 bar); area under the curve (AUC(r), AUC(l)): AUC = $\sum_{i=t1}^{i=t2}U_{i}$ and pre-and post-breathing frequency (Fpre, Fpost): breath/min.

As an index for variations between different experiments the variation coefficient *CV* was calculated using the equation:

$$CV\left[\%\right]=\frac{SD}{median}\times 100$$

### 2.2. Ex Vivo Procedure of Preparation of the Isolated Perfused Rat Lung

**Figure 1:**
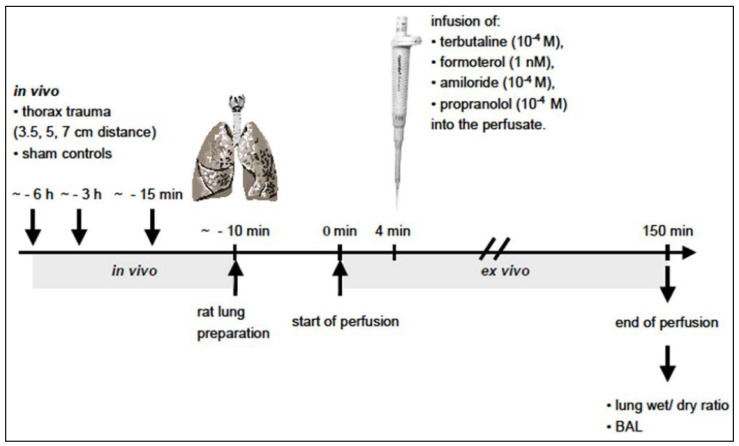
Experimental design of the *ex vivo* studies after *in vivo* thorax trauma.

Figure 1 illustrates the experimental design of the *ex vivo* study following the *in vivo* blast lung injury. A tracheotomy was performed, and the animals were positive pressure ventilated through a tracheal cannula. After thoracotomy and exsanguination, a catheter was placed in the pulmonary artery and the left atrium, and non-recirculating perfusion was started to remove the blood from the pulmonary circulation. The lung was excised, examined macroscopically for areas of pleural and subpleural bleeding using an injury score (Section 2.1.1), and then suspended in the artificial thorax chamber (38 °C). Lung weight was continuously assessed by a weight transducer integrated into the chamber lid [23].

#### 2.2.1. Determination of a Haemorrhage and Oedema Score

The lung lobes were examined macroscopically for areas of pleural and subpleural bleeding using an injury score of 0: no haemorrhage, 1: <30% haemorrhage, 2: 30–60% haemorrhage and 3: >60% haemorrhage. The total score resulted from the summation of the scores of each lobe, but the score of the upper left lobe (≈double size) was duplicated. The inter-observer agreement was >93%. Analogous to the haemorrhage score an oedema score was defined depending on the amount of macroscopically visible oedematous area, using the same scale score. Inter-observer agreement was >90%. Lung wet weight (LWW (g)) and lung wet weight to body weight ratio (LWR) were determined. The mean LWR for the animals exposed to blast injury was expressed as a multiple of the LWR of non-traumatized controls; this ratio was called the injury quotient (Qi). Thus, Qi represents LWR (injured animals) divided by LWR (controls). The extent of the injury was determined as minor or uninjured for Qi < 1.2, moderate for Qi = 1.2 to 1.5, severe for Qi = 1.5 to 1.9, and very severe for Qi > 1.9 as described by Cooper et al. [24].

#### 2.2.2. Experimental Setup

The technical equipment was purchased from Hugo Sachs Electronics-Harvard Apparatus GmbH, Germany, using a standard setup [25]. Excised lungs were perfused at constant hydrostatic pressure (14 cm H_2_O) in a recirculating fashion, with a total volume of about 100 mL. A Krebs–Henseleit perfusion buffer (305–315 mosm, 37 °C) containing 2% bovine serum albumin (BSA), 0.1% glucose and 0.3% HEPES was used. The pH of the perfusate before entering the lung was kept at 7.35 by automatic bubbling of the buffer with carbon dioxide as soon as the pH exceeded this value. The perfusate flow (NarcomaticTM RT 500, Narco Biosystems, TX, USA), the arterial pressure PA, the venous pressure PV (both Isotec™ transducer, Quest Medical, Dallas, TX 75251, USA) and pH (WTW pMX 3000) were continuously monitored. The suspended lungs were ventilated by the trachea with a rotary vane compressor pump (ventilation control module VCM) with negative pressure and ambient air. The breathing frequency was 80 breaths/min. The pulmonary end-expiratory (PEEP), inspiratory (PIP) pressures were set to 2 and 7 cm H_2_O reaching a Tv between 1.8–2.2 mL in untreated control lungs. To avoid the generation of atelectasis as well as to recruit collapsed lung areas hyperinflation of −16 cm H_2_O has triggered automatically every 5 min (timer counter module, TCM). Pulmonary ventilation pressure was measured with a differential pressure transducer (Validyne DP 45-24, Northridge, CA, USA) and airflow velocity was measured with a pneumotachograph connected to the differential pressure transducer (Validyne DP 45–14, Northridge, CA, USA).

Data were transmitted to a computer and analysed by specific software (Pulmodyn software, Hugo Sachs, Marchstetten, Germany): pulmonary pressure (*P*), tidal volume (*Tv*), airway resistance (*R_L_*), dynamic lung compliance (*C_L_*), perfusate flow (*Q*), pulmonary arterial pressure (*P_A_*), pulmonary vein pressure (*P_V_*), vascular resistance (R_V_), perfusate temperature, perfusate pH and lung weight (G). For the calculation of lung mechanics, the following equation was used:

(1) $$P=\left(\frac{1}{C_{L}}\right)T_{v}+R_{L}\left(\frac{dTv}{d_{t}}\right);$$

*t* = time (2)

Vascular resistance (RV) was calculated from the equation:

(3) $$\frac{\left(P_{A}-P_{v}\right)}{Q}$$

In order to obtain minimal ventilation of 1 ml in injured lungs, it was necessary to increase the standard ventilation pressure, depending on the extent of blast injury. The injured lungs were perfused for 150 min just as the control lungs. After the lungs were fixed in the artificial thorax chamber, the ventilation and perfusion parameters were controlled and adjusted. Four minutes after the start of perfusion the lungs were perfused either as controls, without any substance addition, or with addition of terbutaline (10^−4^ M), formoterol (1 nM), amiloride (10^−4^ M), terbutaline + propranolol (10^−4^ M) and terbutaline + amiloride, respectively. The setup of the isolated perfused rat lung is shown in Figure S1.

### 2.3. Statistics

Data in the figures are given as mean and SEM, data in the tables as mean ± SD. The entire curve data from two experiments were compared by a two-way ANOVA design. This test determines how a response is affected by different factors, e.g., drug treatment. Values of *p* < 0.05 were considered statistically significant. Data from end or other time points were analysed by one-way ANOVA. In case of differences among the groups, post-tests were performed as indicated in the legends (Dunnet’s multiple comparison test, Tukey’s multiple comparison test or Bonferroni’s multiple comparison test); statistics were performed with the Graph Pad Prism 3.0 software (Graph Pad Software, Version 6, San Diego, CA, USA).

## 3. Results

### 3.1. Blast Wave Characteristics

A well-defined pressure wave exposure requires a standardized rupture of the polyester diaphragm of the blast wave generator. Utilising the device has been described in our previous work, a complete rupture of the polyester film was reached in all cases. As exemplarily presented in Figure S2, the pressure measurement by the transducer in the reservoir revealed a fast pressure increase of up to 8.7 bar within 19.2 ms, depending on and limited by the opening time of the valve, and an immediate decay to zero indicating a rapid rupture of the diaphragm and therefore a fast exposure of the pressure wave by the nozzle, and a negative phase or suction.

Pressure curves over time were measured by the transducers located on the right and left sides of the rat. At a distance of 3.5 cm of the nozzle to the thorax revealed a nearly instantaneous 100% rise time of 85 and 295 µs to the pressure peaks of 3.4 and 3 bar, and a duration of 654 and 625 µs, respectively (Figure S3). The calculated area under the curves (AUC (r), AUC (l)) were 101.35 and 100.37 V × s.

Table S2 summarises the pressure wave data and the calculated area under the curves at a 3.5 cm distance to the blast source measured in the present study. The mean values of the pressure wave data measured by the right and left transducer (3.16 ± 0.43 bar, duration 628.78 ± 29.31 µs, *n* = 219) were assumed to be the pressure wave characteristics obtained above the animal’s thorax.

The mean pressure peak measured at the animals’ thorax level decayed rapidly as it expanded from the blast wave generator. This pressure decreases correlated significantly (r = −0.58) with the distance, allowing animals’ exposure to different blast intensities by simply varying the distance between the blast wave generator and the animal. Moreover, the mean duration of the pressure wave and therefore the impact on the rat and the calculated area under the curve also correlated significantly with the distance (r = 0.24 and r = 0.51, respectively) (Figure S4).

### 3.2. Interrelationships between Physical and Physiological Injury

**Figure 2:**
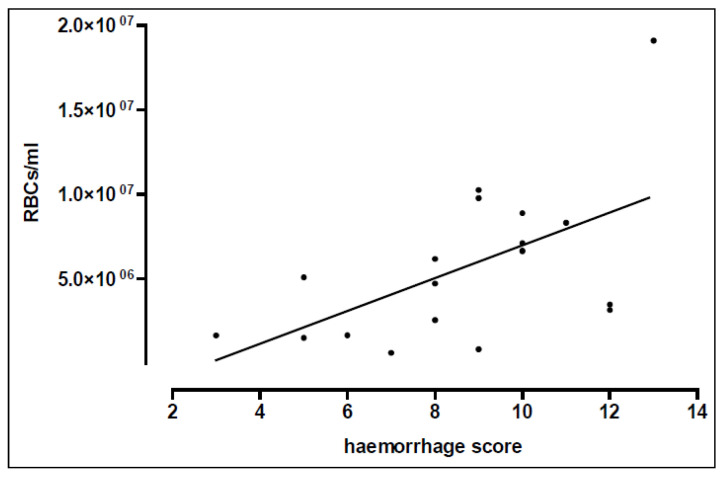
Distance-dependent alveolar haemorrhage. Red blood cells (RBCs)/haemorrhage score correlation. The animals were located either 3.5 (*n* = 12), 5 (*n* = 4), or 7 cm (*n* = 3) distance from the nozzle, non-traumatized animals served as sham controls (*n* = 5). Statistical analysis was performed on (A) by One-way ANOVA and Dunnett’s multiple comparison test: *p* < 0.01 vs. sham control. Linear regression revealed a Pearson r of r = −0.49, *p* = 0.033. Data are expressed as mean ± SD, number of experiments (*n*) and (B) by correlating RBCs with the score and linear regression revealing a Spearman r of r = 0.565.

The animals were subjected to a blast pressure wave with the tip of the blast nozzle located either 3.5, 5, or 7 cm above the sternum. The alveolar haemorrhage measured directly after trauma and expressed as red blood cells per ml BAL fluid demonstrated a marked increase in rats positioned at a 3.5 cm distance below the nozzle, as compared to non-injured control lungs (*p* < 0.01) (Figure 2, Table 1).

Furthermore, the lung wet weight/body weight ratio (LWR), was significantly increased in animals placed at 3.5 cm distance below the nozzle in comparison to sham controls and animals at 7 cm distance (*p* < 0.05) (Table 2). Comparison of the calculated injury quotients as described by Cooper et al. [24] revealed a quotient of 1.94 for the 3.5 cm group, 1.31 for the 5 cm group and 1.20 for the 7 cm group, indicating “very severe“, “moderate” and “minor” lung injury, respectively (Table 2). In addition, the individually defined haemorrhage and oedema scores showed significantly higher values in the 3.5 and 5 cm group compared to non-traumatized sham controls (*p* < 0.001; *p* < 0.01) and in the 3.5 cm group compared to the 7 cm group (*p* < 0.05) (Table 2).

### 3.3. Lung Function

**Figure 3:**
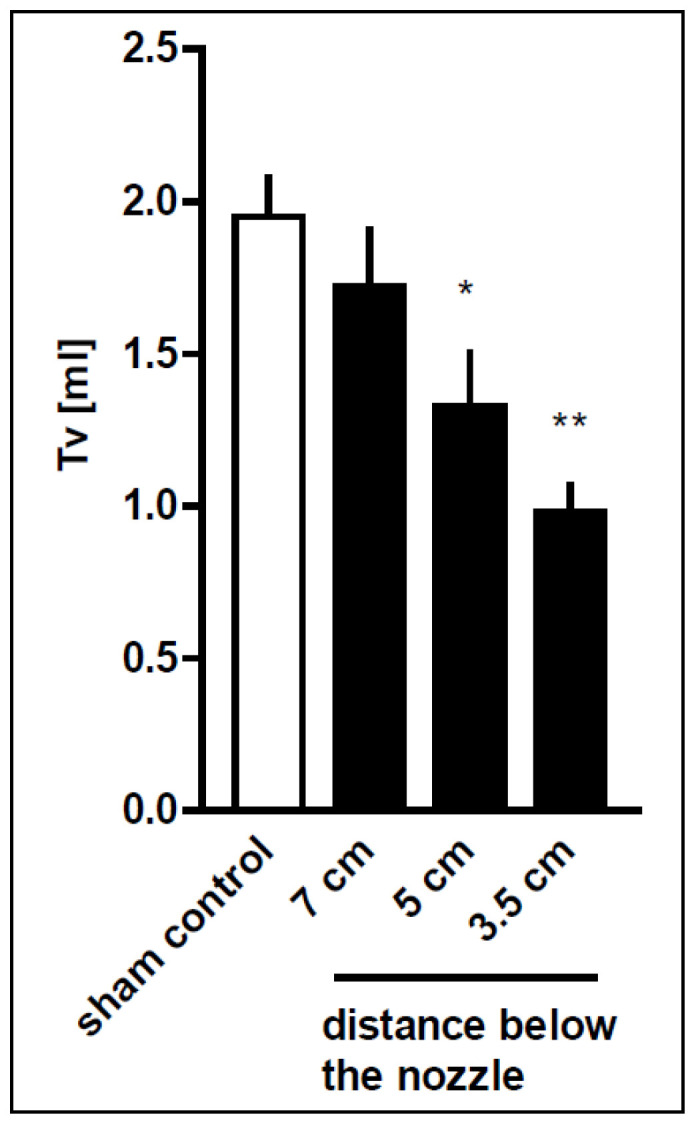
Pressure wave intensity-related decrease in initial tidal volume after thoracic trauma. Rats were exposed to the pressure wave at different distances from the nozzle (3.5 cm, *n* = 9; 5 cm, *n* = 16; 7 cm, *n* = 7). The rat lungs were perfused immediately after blast. Non-traumatized animals served as sham controls (*n* = 7). The initial tidal volume (Tv) was assessed with standard ventilation pressures (PEEP/PIP: −2/−7 cm H_2_O). Data are expressed as mean ± SD, number of experiments (*n*). Statistical analysis was performed by one-way ANOVA and Dunnett’s multiple comparison test: * *p* < 0.05 and ** *p* < 0.01 vs. sham control. Correlation between distance and Tv was performed by linear regression revealing a Pearson r of r = 0.58, *p* = 0.0005.

The initial tidal volume and therefore the compliance of the lungs immediately after blast injury, obtained with standard ventilation pressures (PEEP/PIP: 2/7 cm H_2_O) were significantly decreased in the 3.5 cm nozzle distance group (0.98 ± 0.35 mL) (*p* < 0.01) and the 5 cm distance group (1.33 ± 0.52 mL) as compared to the sham controls (1.95 ± 0.33 mL; *p* < 0.05) (Figure 3). The decrease in the initial tidal volume correlated with the distance below the nozzle (r = 0.58, *p* < 0.001).

When adjusting the ventilation pressures in order to obtain minimal ventilation of 1 mL tidal volume the rats exposed to blast pressure injury at a distance of 3.5 cm required a significantly higher inspiratory pressure (8.36 ± 1.36 cm H_2_o) in comparison to sham controls (7.0 cm H_2_o) (*p* < 0.05) (Table 3). Lung function parameters of injured rat lungs measured after a time span of 150 min perfusion were significantly deteriorated in the 3.5 cm distance group, as compared to non-traumatized controls with respect to tidal volume (0.66 ± 0.21 mL vs. 1.60 ± 0.15 mL; *p* < 0.001), airway resistance (0.47 ± 0.15 cm H_2_O/L/s vs. 0.33 ± 0.05 H_2_O/L/s; *p* < 0.05) and lung weight gain (1099 ± 441 mg vs. 579 ± 184 mg; *p* < 0.01) (Table 3). Measurement of vascular parameters (pulmonary arterial pressure, pulmonary vein pressure, vascular resistance) did not reveal significant differences.

**Figure 4:**
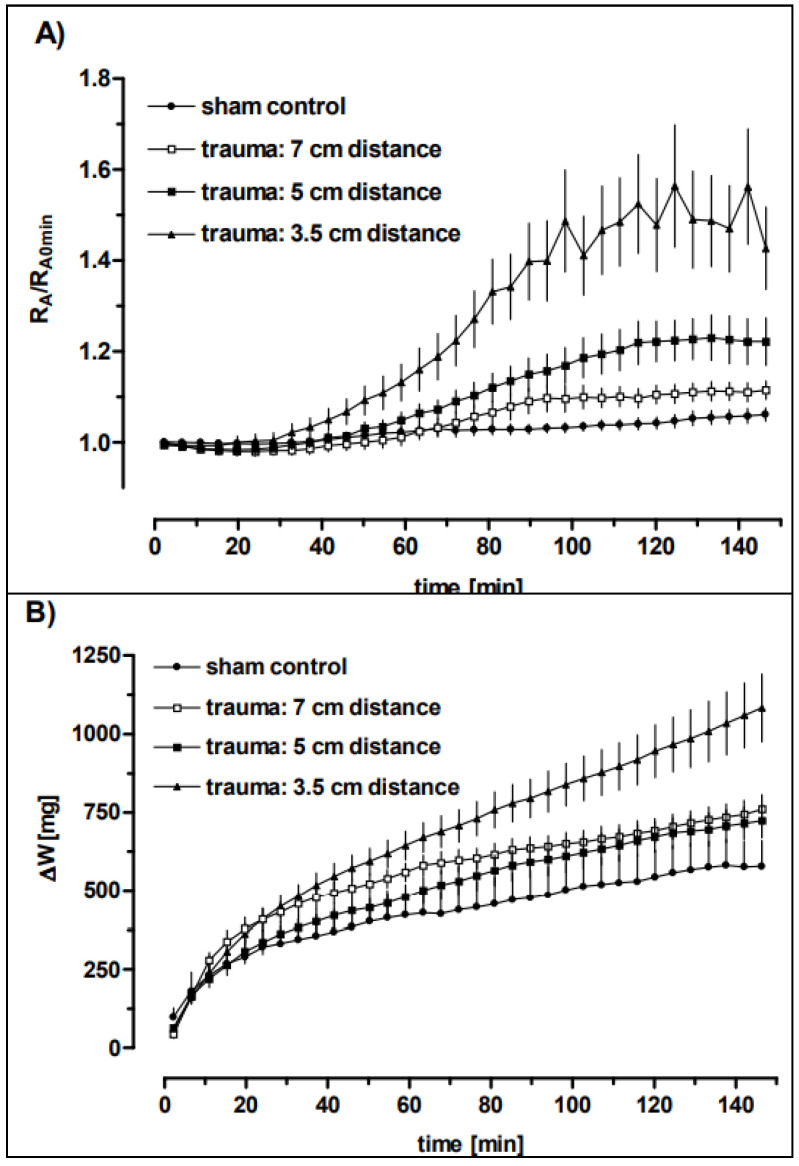
Bronchoconstriction and pulmonary oedema *ex vivo* as a function of distance between rat thorax and blaster nozzle. Rats were exposed to the pressure wave at different distances from the nozzle (3.5 cm, 5 cm, and 7 cm). Immediately after blast the lung perfusion was started. Nontraumatised animals served as sham controls. Data are expressed as mean and SEM (given in part as ± SEM, in part as + SEM). (**A**) R_A_/R_A0_ min: airway resistance and (**B**) ΔW: weight gain were all normalized to the value at t = 0 min.

The comparison of the airway resistance and the lung weight gain of the differently injured groups revealed a distance dependency (Figure 4 and Table 3). This was not the case for the tidal volume. Despite this, at the end of perfusions, no distance dependency could be measured.

### 3.4. Time-Dependent Recovery of the Lung Function after Trauma

We further investigated whether the pulmonary oedema formation and deterioration of the lung function observed *ex vivo* initially after the blast was also time-dependent. Therefore, the animals were subjected to a blast wave injury at a distance of 3.5 cm and sacrificed at ten minutes, three hours and six hours after trauma. Subsequently, the lungs were perfused for 150 min. The red blood cell counts examined after 150 min perfusion time did not significantly differ within the three groups (10 min: 7.13 ± 4.72 × 10^6^/mL, *n* = 12; 3 h: 6.74 ± 2.71 × 10^6^/mL, *n* = 4; 6 h: 10.9 ± 7.65 × 10^6^/mL, *n* = 3).

**Figure 5:**
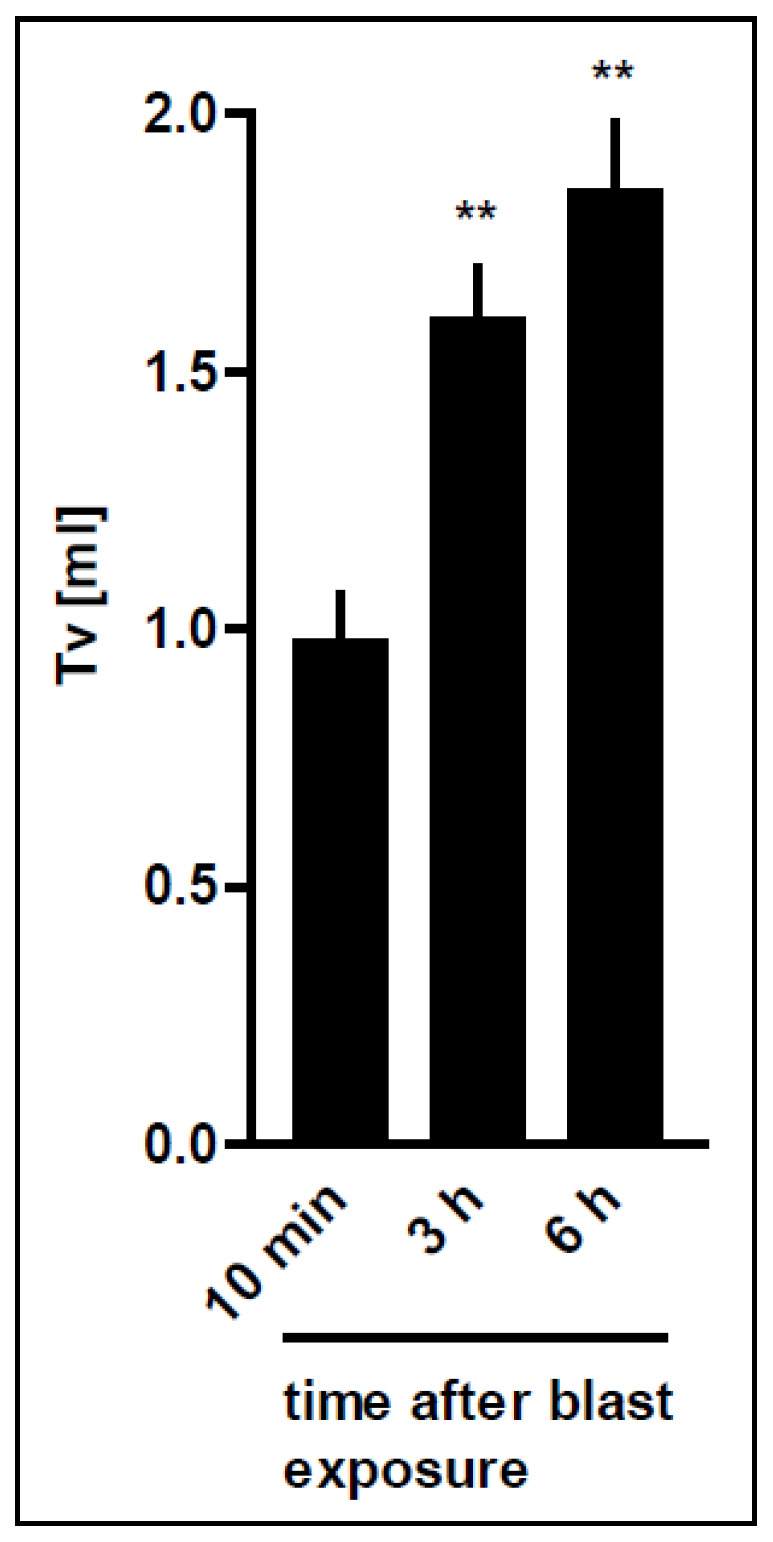
Time-dependent improvement of tidal volume after thoracic trauma. Rats were exposed to the pressure wave at a distance of 3.5 cm from the nozzle. The rats were sacrificed either 10 min (*n* = 16), 3 h (*n* = 7) or 6 h (*n* = 7) following the blast. Subsequently, lung perfusion was started. The initial tidal volume (Tv) was assessed at standard ventilation pressures (PEEP/PIP: −2/−7 cm H_2_O). Data are expressed as mean ± SD, number of experiments (*n*). Statistical analysis was performed by one-way ANOVA and Dunnett’s multiple comparison test: ** *p* < 0.01 vs. 10 min. Correlation between time after blast and Tv was performed by linear regression revealing a Pearson r of r = 0.74, *p* < 0.0001.

Linear correlation showed that the initial tidal volume obtained with standard ventilation pressures (PEEP/PIP: 2/7 cm H_2_O) was improved depending on the time elapsed after blast exposure (r = 0.74). Thus, the initial tidal volume at the beginning of *ex vivo* perfusion was significantly improved 3 and 6 h after blast exposure, as compared to perfusion immediately after the blast pressure injury (*p* < 0.0001) (10 min: 0.98 ± 0.35 mL; 3 h: 1.60 ± 0.29 mL; 6 h: 1.85 ± 0.35 mL) (Figure 5). Within six hours after the blast, the tidal volume recovered and reached sham control levels (1.95 ± 0.33 mL), indicating that some kind of repair takes place *in vivo* over time. These data are in line with the improved compliance over time.

Due to this time-dependent alteration of the respiratory mechanics following blast injury, the necessary inspiratory pressure (PIP) to achieve 1 mL tidal volume was significantly reduced 3 and 6 h after the blast as compared to immediately perfused lungs (*p* < 0.01 and *p* < 0.05, respectively). Despite the significantly improved initial tidal volume in lungs perfused three and six hours after trauma compared to immediately perfused lungs (10 min), no significant difference in tidal volume or in airway resistance between the groups could be measured at the end of perfusion (Table 4).

**Figure 6:**
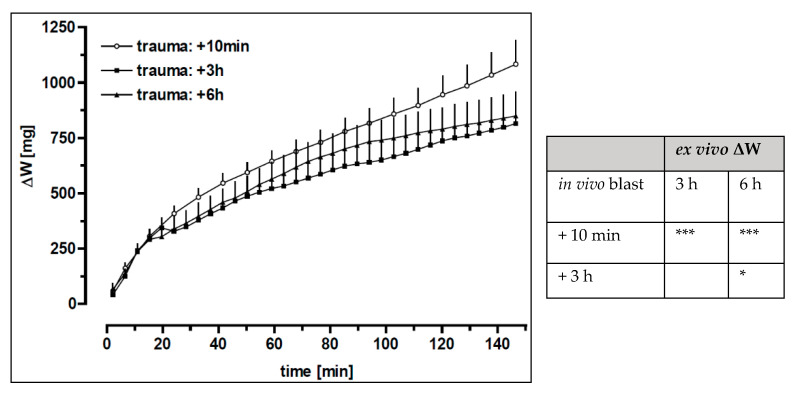
Pulmonary oedema *ex vivo* as a function of time Rats were exposed to the pressure wave at 3.5 cm from the nozzle. The rats were sacrificed either 10 min (*n* = 15), 3 h (*n* = 8) or 6 h (*n* = 8) following the blast. Subsequently, lung perfusion was started. Data are expressed as mean + SEM. Lung weight gain ΔW: was assessed over 150 min of perfusion time. Statistical analysis of the entire curve data from different groups was compared by a two-way ANOVA design. The *p*-value represents the time-dependent recovery after trauma: * *p* < 0.05 and *** *p* < 0.0001.

Comparison of the lung weight data revealed an enhanced weight gain of the lungs immediately perfused after injury, as compared to lungs perfused 3 or 6 h after blast (*p* < 0.0001) (Figure 6). However, analysis of the lung weight values assessed at the end of perfusion (Table 4) revealed no significant differences between the groups. The macroscopically defined oedema score correlated with the time after blast exposure (r = −0.85) and was significantly increased in recently injured lungs in comparison to lungs after a time span of 3 and 6 h after the blast, respectively (*p* < 0.0001) (10 min: 10.1 ± 2.4, *n* = 16; 3 h: 1.1 ± 1.9, *n* = 8; 6 h: 0.6 ± 1.5, *n* = 7), thus suggesting a recovery *in vivo* over time.

### 3.5. Pharmacological Interventions

#### 3.5.1. The Role of the Amiloride-Sensitive Sodium Channels after Trauma

Amiloride blocked the terbutaline-related decrease in lung weight (Figure 6 and Table 5), indicating that β_2_-receptor-related stimulation is dependent on amiloride-sensitive sodium transport, as also proposed by several publications [26,27]. However, statistical analysis by two-way ANOVA yielded also a significant difference between terbutaline plus amiloride versus amiloride treatment alone, suggesting the implication of amiloride-insensitive sodium transport. As expected, and in contrast to the weight gain, amiloride did not influence the terbutaline-induced improvement of the tidal volume (Tv), airway resistance (R_A_) (Figure 6 and Table 5), which thus may be considered a sodium channel-independent β_2_-adrenergic effects.

**Figure 7:**
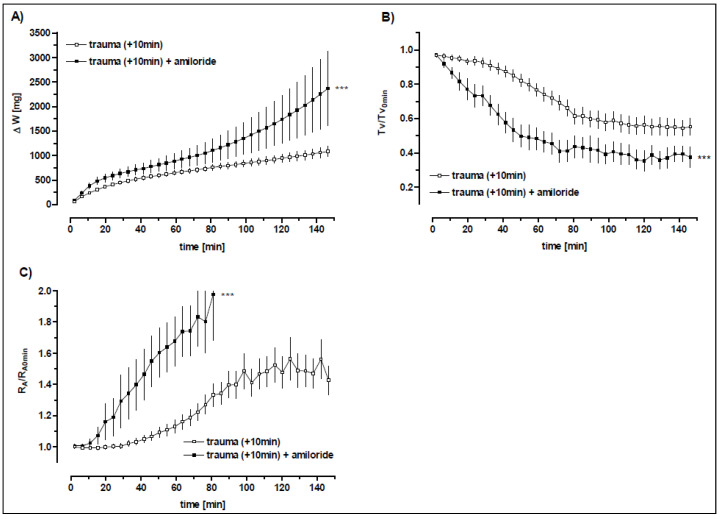
**Role of sodium channels after trauma.** Rats were exposed to the pressure wave at 3.5 cm from the nozzle. The rats were sacrificed 10 min (*n* = 24) after the blast. The lungs were treated either with (*n* = 8) or without (*n* = 16) amiloride (10^−4^ M) 4 min after the start of perfusion. Data are expressed as mean ± SEM. (**A**) ΔW: weight gain, (**B**) Tv/Tv_0 min_: normalized tidal volume and (**C**) RA/RA_0 min_: normalized airway resistance. Statistical analysis of the entire curve data from two groups were compared by a Two-way ANOVA design: *** *p* < 0.0001.

Post-blast perfusion of the lung with the addition of amiloride resulted in a further increase in oedema formation in comparison to untreated blast-injured lungs as indicated by weight gain (*p* < 0.001). Similarly, tidal volume was significantly diminished (*p* < 0.001) and airway resistance significantly increased (*p* < 0.001) in amiloride-treated animals (Figure 7; Table 5).

#### 3.5.2. β_2_-Adrenergic Receptor Stimulation

**Figure 8:**
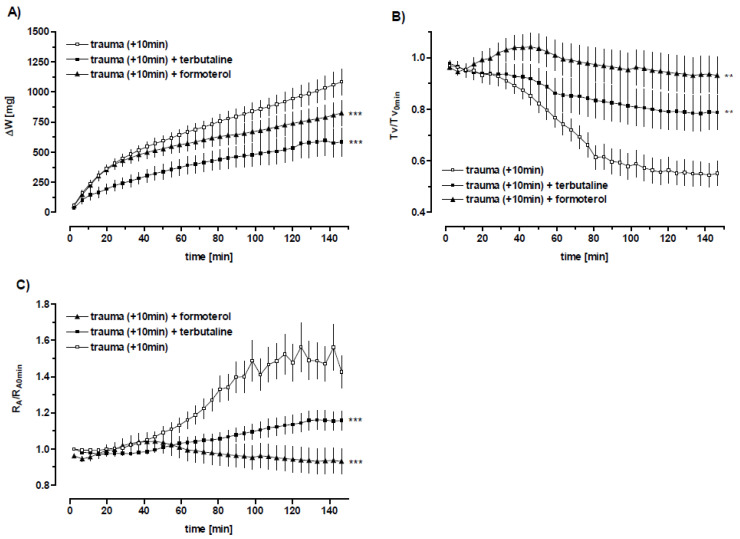
Modulation of the trauma-induced pulmonary dysfunction by terbutaline and formoterol. Rats were exposed to the pressure wave at a distance of 3.5 cm from the nozzle. The rats were sacrificed 10 min (*n* = 37) following the blast. The lungs were perfused either untreated (*n* = 16) or treated with terbutaline (100 µM) (*n* = 9) and formoterol (1 nM) (*n* = 12), respectively, 4 min after start of perfusion. Data are expressed as mean ± SEM. (**A**) ΔW: weight gain, (**B**) Tv/Tv_0_ min: normalized tidal volume and (**C**) R_A_/R_A0 min_: normalized airway resistance were monitored over 150 min perfusion time. Two-way ANOVA analysis: ** *p* < 0.01, *** *p* < 0.0001 vs. trauma (+10 min) (Table 5).

In these experiments, thoracic trauma-related deterioration of the lung parameters was markedly improved by terbutaline and formoterol with respect to tidal volume (T_v_), airway resistance (R_A_) and weight gain (∆W) (Figure 8A–C, Table 5 and Table 6). With regard to the weight gain of blasted rat lungs during perfusion, the terbutaline-induced reduction reached control levels of non-traumatized lungs (∆W: 559 ± 368 mg and 579 ± 184 mg for traumatized rat lungs plus terbutaline treatment and sham controls, respectively) (Table 5). Taken together, these data indicate that β_2_-adrenoceptor agonist treatment improves the lung weight and the lung function parameters after blast exposure. The terbutaline-induced amelioration in T_v_ and R_A_ was completely blocked by the non-specific β-receptor antagonist propranolol (Table 5 and Table 6). The oedema formation was even more impaired than in non-treated blasted rat lungs (Table 5 and Table 6).

#### 3.5.3. The Impact of the Formoterol after Trauma

Adding formoterol to the perfusate led also to a significant increase in the tidal volume (*p* < 0.001) and a significant decrease in the resistance (*p* < 0.01) compared to untreated blast-injured lungs, while the lung weight was not influenced. The terbutaline-induced reduction of weight gain and the decrease in airway resistance were completely blocked by the non-specific β-receptor antagonist propranolol (*p* < 0.05) (Table 5). Similarly, amiloride added to terbutaline perfusion reversed terbutaline-induced weight gain reduction (*p* < 0.05), while tidal volume and resistance were not significantly influenced (Table 5). However, tidal volumes of blasted and thereupon terbutaline-amiloride perfused lungs were significantly higher than tidal volumes after trauma and amiloride perfusion alone (*p* < 0.05) (Table 6) indicating a sodium channel-independent β_2_ -adrenergic effect.

## 4. Discussion

The study aimed to investigate the role of blast-induced pulmonary oedema generation as a function of time and trauma intensity and the role of β_2_-adrenoceptor agonists on oedema, assessed in an *ex vivo* setting. The experiment started with *in vivo* blast-induced thorax trauma and was followed by analysing of *ex vivo* isolated perfused rat lung. If added as a control amiloride to the perfusion, it caused more than a double-fold increase in oedema formation during perfusion in blasted lungs. Accordingly, it indicates partial preservation of the sodium channels and therefore, a fluid reabsorption capacity of them after thorax trauma. Inhibition of the endogenous fluid resorption in traumatized rat lungs by amiloride furthermore resulted in a marked deterioration of the airway mechanics. This may be due to the oedema formation rather than to other further pharmacological effects of amiloride.

Upon administration of terbutaline or formoterol in the perfusate of the blast group, the weight gain during perfusion was significantly reduced comparing the blast trauma only group. Interestingly, upon terbutaline treatment, virtually control levels of non-traumatized rat lungs were reached. In rats, the response to β_2_-adrenoceptor agonist included an increase in sodium unidirectional flux out of the alveoli and stimulation of fluid absorption. Terbutaline improves ischemia-reperfusion injury after left-sided orthotopic rat lung transplantation [28]. Apparently, terbutaline stimulates the transport through amiloride-sensitive pathways since amiloride reduced the terbutaline-related fluid resorption in the current setting. This result is in accordance with prior studies depicting the effect of amiloride on the terbutaline-induced increase in alveolar liquid clearance.

However, infusion of amiloride plus terbutaline resulted in decreased oedema formation in this model compared to amiloride treatment alone. The most reasonable explanation for this finding is that terbutaline stimulates sodium transport through both amiloride-sensitive and insensitive pathways. Similar results have been reported previously. In the isolated perfused rat lung, amiloride alone reduced sodium transport by 30%, terbutaline increased it by 59%, but terbutaline plus amiloride decreased clearance only by 14%. Another explanation could be the permeability decreasing the action of terbutaline, leading to a reduction of oedema generation after trauma. It has been reported that isoproterenol as a non-selective β adrenoceptor agonist attenuates the thrombin-induced increase in endothelial permeability *in vitro* [29].

The experiments with propranolol indicate that the effect of terbutaline on airway mechanics in blasted rat lungs is mediated through β-receptors. However, oedema formation and vascular resistance was even more impaired upon terbutaline plus propranolol treatment, as compared to non-treated blasted rat lungs. The block of β_2_-receptors causes well-known untoward effects, e.g., vasoconstriction, delayed response to hypoglycaemia in diabetic patients, and bronchoconstriction, which could be involved in the observed effects. However, the basal β-adrenergic tone, described in anaesthetised rats [30] is probably not an important factor in the normal fluid clearance, because propranolol alone did not have an effect on lung liquid clearance in this model.

β_2_-adrenergic intervention not only reduced oedema generation after trauma, but also improved the airway mechanics, possibly by direct β_2_-adrenergic effects that may be independent of β-receptor-related fluid resorption. Trauma-related increase in the airway resistance was markedly reduced by β_2_-adrenoceptor agonists treatment. The reduced airway resistance may be due to the β_2_-receptor-mediated airway smooth muscle relaxation but also by fluid shifts, by a β-agonist-related modulation of inflammation, or by modulation of pulmonary surfactant synthesis.

Thus, β_2_-adrenergic substances appeared to be beneficial in oedema generation in this model. It has been shown that β_2_AR contributes to the β-adrenergic–mediated upregulation of alveolar fluid clearance [31]. β_2_-adrenoceptor agonists via activation of β_2_ARs regulate necessary key proteins for the process of alveolar epithelial active Na^+^ transport through activation and increase in expression of ion channels and reduce pulmonary endothelial permeability [31,32,33,34,35,36]. Ion channels which can be modulated by β_2_-adrenoceptor agonists by their activity or expression include epithelial sodium channel (ENaC) [21,36,37], Na^+^/K^+^-ATPase [30,38,39,40,41], cystic fibrosis transmembrane conductance regulator (CFTR) [42], cyclic nucleotide-gated cation (CNG) channels [21], nonselective cation channel (NSCCa) [43] and Ca^2+^ dependent K^+^ channel (BKCa) [44,45]. Moreover, treatment with β2-agonists increases the secretion of surfactant proteins and exerts anti-inflammatory properties by influencing several types of immune cells and reducing fibrotic remodelling [16,46]. However, β_2_-adrenoceptor agonists have not been shown to be beneficial in two clinical studies of acute respiratory distress syndrome (ARDS) that included multiple causes of lung injuries [36]. On the other hand, high-altitude oedema could be prevented by the use of the β_2_ receptor agonist salmeterol [47]. In a recent explorative randomised study, five days of inhaled budesonide/formoterol in patients at risk for ARDS has improved patient oxygenation [48], and similar studies aiming at early intervention are ongoing in SARS-CoV-2 infection to prevent lung injury.

Further biological factors may restrict the β_2_-adrenoceptor agonist use to increase the resolution of pulmonary oedema [49]. Prolonged stimulation of β-adrenergic receptors with endogenous catecholamines may desensitize the β-receptors and prevent their stimulation with exogenous agonists [49]. In some patients, the alveolar epithelium may be too injured to respond to β-adrenergic agonist therapy [49], possibly also by circulating or local factors, e.g., in the alveolar space that could limit the action of β_2_-adrenoceptor agonist [49]. In addition, in the presence of left atrial hypertension, atrial natriuretic peptide (ANP) can inhibit the stimulatory effect [49]. Similarly, in prolonged haemorrhagic shock and resuscitation, cAMP agonists may not stimulate AFC because of an oxidant-mediated injury that may reduce the alveolar epithelium to β_2_-adrenoceptor agonist response [49].

A further clinical aspect is the potential to increase the cardiac index by β_2_-adrenoceptor agonist [50], through both cardiac stimulation and pulmonary arterial vasodilation. Cardiac stimulation can lead to a higher cardiac index. This is potentially dangerous, as due to the injured lung put in the circulation in series, there is an increased infiltration, which further increases alveolar fluid and gas exchange disturbance. An interrelated second, and in ALI most probably untoward “Robin Hood effect” of the potential opening of vascular beds that are closed by vasoconstriction is, e.g., observed in COPD patients inhaling β_2_-adrenoceptor agonist and developing more hypoxemia [51]. This is presumably due to increased perfusion in inappropriately ventilated ALI/ARDS alveolar areas. As shown by Briot et al., β_2_-adrenoceptor agonist therapy seems, therefore, to have the potential to heighten the protein leakage from plasma to alveoli in the acutely injured lung *in vivo* [50].

Of high interest would be alternative oedema reabsorption and barrier-tightening function studies that target alveolar liquid clearance. One focus is ENaC stimulation, e.g., by the lectin-like region of TNF that stimulates ENaC and is protective for the alveolar endothelial–epithelial barrier and anti-inflammatory [28,36,52].

Limitations of our study are the use of one dose for studied substances, and limited time points, due to the high complexity, resources, and ethical pressure to especially limit animal research. Inter-species differences, size [53], the complexity of blast physics and animal-to-human scaling issues [54] further keep the difficulty in translating studies into clinical medicine.

In conclusion, the combined *in vivo*–*ex vivo* rat lung oedema assessment gave clear evidence of the time and dose relationship of oedema and of the amiloride-dependent beta-2-adrenoceptor oedema reduction. However, alternative ENaC stimulating substances may be of more scientific interest, as clinical studies using beta-2 agonists in ARDS patients have failed [36].

## Figures and Tables

**Table 1:** Selected results from exposure to graded blast waves.

Distance	RBCs × 10^6^/mL	Haemorrhage Score	Oedema Score
sham control	0.12 ± 0.15 (5)	0 ± 0 (5)	0 ± 0 (4)
3.5 cm	7.13 ± 4.72 (12) **	9.6 ± 2.2 (12) ***	10.0 ± 3.6 (3) ***
5 cm	4.68 ± 3.11(4)	8.3 ± 2.1 (4) ***	7.2 ± 1.9 (5) **
7 cm	1.33 ± 0.44 (3)	5.7 ± 3.1 (3) ** #	4.0 ± 1.4 (3) #

One-way ANOVA and Tukey’s multiple comparison test: ** *p* < 0.01, *** *p* < 0.001 vs. sham control; # *p* < 0.05 vs. 3.5 cm. RBCs: red blood cells. Data are expressed as mean ± SD, and number of experiments is mentioned in brackets (*n*).

**Table 2:** Lung wet weight to body weight ratio at varying distances from the nozzle.

Distance	*n*	BW [g]	LWW [g]	LWR	Qi
sham control	4	269 ± 30	1.83 ± 0.13	0.0069 ± 0.0013	
3.5 cm	3	243 ± 6	3.21 ± 1.25 *	0.0130 ± 0.0053 *	1.94
5 cm	5	241 ± 6	2.14 ± 0.23	0.0089 ± 0.0009	1.31
7 cm	3	238 ± 12	1.94 ± 0.06 #	0.0082 ± 0.0006 #	1.20

One-way ANOVA and Tukey’s multiple comparison test: # *p* < 0.05 vs. 3.5 cm and * *p* < 0.05 vs. sham control. Data are expressed as mean ± SD, and the number of experiments is mentioned in brackets (*n*). BW: body weight; LWR: lung wet weight to body weight ratio; LWW: lung wet weight; Qi: injury quotient.

**Table 3:** Thoracic trauma-induced evolution of lung function parameters.

*In Vivo* Trauma	*Ex Vivo* Perfusion			
~−15 min	~3 min	150 min	150 min	150 min
Distance	PIP (cm H_2_O)	T_v_ (mL)	R_A_ (cm H_2_Oxs/mL)	ΔW (mg)
Sham control	7 ± 0 (7)	1.60 ± 0.15 (7)	0.33 ± 0.05 (7)	579 ± 184 (6)
3.5 cm	8.36 ± 1.36 * (16)	0.66 ± 0.21 *** (13)	0.47 ± 0.15 * (15)	1099 ± 441 ** (15)
5 cm	7.31 ± 0.64 # (11)	0.68 ± 0.23 *** (11)	0.34 ± 0.05 # (10)	723 ± 158 # (9)
7 cm	7.07 ± 0.07 # (7)	0.90 ± 0.31 *** (7)	0.29 ± 0.02 ## (7)	789 ± 114 (6)

Rats were exposed to the pressure wave at different distances from the nozzle (3.5 cm, 5 cm, and 7 cm). Immediately after the blast, lung perfusion was started, and the lung function parameters were recorded. Non-traumatized animals served as sham controls. Inspiratory pressure (PIP), tidal volume (T_v_), airway resistance (R_A_) or weight gain (ΔW). Data are expressed as mean ± SD, the number of experiments (*n*) in brackets. One-way ANOVA and Tukey’s multiple comparison test were performed: * *p* < 0.05, ** *p* < 0.01, *** *p* < 0.001 vs. sham control, and # *p* < 0.05, ## *p* < 0.01 vs. 3.5 cm, respectively.

**Table 4:** Lung function assessed *ex vivo* at different time points after blast-related injury.

*In Vivo* Trauma	*Ex Vivo* Perfusion			
~−15 min	0 min	150 min	150 min	150 min
Time after blast	PIP (cm H_2_O)	Tv (mL)	R_A_ (cm H_2_Oxs/mL)	ΔW (mg)
10 min	8.36 ± 1.36 (16)	0.66 ± 0.21 (13)	0.47 ± 0.15 (15)	1099 ± 441 (15)
3 h	7.07 ± 0.11 ** (8)	0.83 ± 0.31 (7)	0.36 ± 0.07 (7)	826 ± 293 (8)
6 h	7.09 ± 0.08 * (8)	0.76 ± 0.34 (6)	0.38 ± 0.09 (6)	855 ± 311 (8)

Rats were exposed to the pressure wave at a distance of 3.5 cm from the nozzle. The rats were sacrificed either 10 min, 3 h or 6 h following the blast. Subsequently, lung perfusion was started. Inspiratory pressure (PIP), tidal volume (T_V_), airway resistance (R_A_), weight gain (ΔW). Data are expressed as mean ± SD, number of experiments (*n*). One-way ANOVA and Dunnett’s multiple comparison test were performed: * *p* < 0.05, ** *p* < 0.01 vs. 10 min.

**Table 5:** Statistical analysis of different pharmacological interventions.

	Tv (mL)			R_A_ (cm H_2_Oxs/mL)			ΔW (mg)		
Treatment	Trauma	+Terbutaline	+Amiloride	Trauma	+Terbutaline	+Amiloride	Trauma	+Terbutaline	+Amiloride
+amiloride	**>0.0005**			**>0.0005**			**>0.0005**		
+terbutaline	**>0.0005**			**>0.0005**			**>0.0005**		
+formoterol	**>0.0005**	**>0.0005**		**>0.0005**	**>0.0005**		**>0.0005**	**>0.0005**	
+terbutaline +propranolol	**ns**	**>0.0005**		**ns**	**>0.0005**		**>0.0005**	**>0.0005**	
+terbutaline +amiloride	**>0.0005**	**ns**	**>0.0005**	**>0.0005**	**ns**	**>0.0005**	**>0.0005**	**>0.0005**	**>0.005**

Statistical analysis of the entire curve data from two groups was compared by a two-way ANOVA design: analysed condition was the pharmacological modulation during perfusion.

**Table 6:** Pharmacological intervention: role of amiloride-sensitive sodium channels and β_2_ adrenergic receptor modulation.

*In Vivo* Trauma	*Ex Vivo* Perfusion			
~−15 min	~4 min	150 min	150 min	150 min
	treatment	Tv (mL)	RA (cm H_2_Oxs/mL)	ΔW (mg)
Sham control		1.60 ± 0.15 (7)	0.33 ± 0.05 (7)	579 ± 184 (6)
Trauma		0.66 ± 0.21 (13) §§§	0.47 ± 0.15 (15) §	1099 ± 441 (15) §
	+ amiloride	0.56 ± 0.09 (4)	1.60 ± 0.46 (5) *** ⊗	2429 ± 2265 (8) *
	+ terbutaline	1.08 ± 0.26 (9) ***	0.33 ± 0.04 (9) **	559 ± 368 (8) **
	+ formoterol	1.12 ± 0.34 (12) ***	0.32 ± 0.04 (12) **	827 ± 381 (12)
	+ terbutaline + propranolol	0.89 ± 0.51 (3)	0.40 ± 0.12 (3) #	3378 ± 2779 (5) ***,#
	+ terbutaline + amiloride	1.09 ± 0.61 (4) *	0.35 ± 0.12 (4)	1534 ± 799 (4) #

Rats were exposed to the pressure wave at a distance of 3.5 cm from the nozzle. The rats were sacrificed 10 min (*n* = 55) following the blast. After preparation of the lungs, the blood-free perfusion was started, and the lung function parameters were recorded. The lungs were perfused either untreated (*n* = 16) or treated with amiloride (10^−4^ M) (*n* = 8), terbutaline (10^−4^ M) (*n* = 9), formoterol (1 nM) (*n* = 12), terbutaline + propranolol (10^−4^ M) (*n* = 5) and terbutaline + amiloride (*n* = 5), respectively, 4 min after start of perfusion. Non-traumatized animals served as sham controls (*n* = 7). Data are expressed as mean ± SD and represent the values assessed after *t* = 150 min perfusion time (except⊗: value at *t* = 80 min). Data were analysed by an unpaired *t*-test: § *p* < 0.05 and §§§ *p* < 0.001 vs. sham control; * *p* < 0.05, ** *p* < 0.01 and *** *p* < 0.001 vs. trauma.

## Supplementary Materials

The following supporting information can be downloaded at: https://www.mdpi.com/article/10.3390/biomedicines10112930/s1, Figure S1. The Setup of the isolated perfused rat lung; Figure S2. Time course of pressure increase in the pressure reservoir until rupture of the diaphragm; Figure S3. Pressure wave monitoring at rat thorax level; Figure S4. Pressure wave intensity-related decrease in initial tidal volume after thoracic trauma; Table S1 Chemicals used in the isolated perfused rat lung experiments and chemicals used in anaesthesia and analgesia; Table S2. Pressure wave data measured by different transducers at various positions.
