# Supplementary material for: Ex Vivo Pulmonary Oedema after In Vivo Blast-Induced Rat Lung Injury: Time Dependency, Blast Intensity and Beta-2 Adrenergic Receptor Role
Source: Biomedicines. 2022 Nov 15;10(11):2930. doi: 10.3390/biomedicines10112930 (PMC9687465; doi:10.3390/biomedicines10112930)
Supplement: Supplementary file 1 [file biomedicines-10-02930-s001.zip › biomedicines-1921243-supplementary.pdf]

Supplementary materials

Material information

Table S1 Chemicals used in the isolated perfused rat lung experiments and chemicals used in anaesthesia and analgesia.

Substance	Producer	Solute in	Concentration	Storage
Amiloride	Sigma-Aldrich, Deisenhofen, Germany	DMSO, NaCl	10^{-4} M	Freshly prepared
Bovine serum albumin fraction V, receptor-grade; Lot No. 12353	Serva, Heidelberg, Germany	buffer	2%	Freshly prepared
Formoterol	AstraZeneca, Zug, Switzerland	saline, 0.05% $Na_2S_2O_5$	1 nM	Freshly prepared
Propranolol	Sigma-Aldrich, Deisenhofen, Germany	saline	10^{-4} M	Freshly prepared
Terbutaline	Sigma-Aldrich, Deisenhofen, Germany	NaCl	10^{-4} M	stock at -20°C
Halothane, 2-bromo-2-chloro-1, 1, 1-trifluoroethane	Sigma-Aldrich Chemie GmbH, Deisenhofen, Germany			
Pentobarbital sodium (Narcoren®)	Wirtschaftsgenossenschaft deutscher Tierärzte, Hannover, Germany			
Buprenorphine (Temgesic®)	Tierforschungsanlage, Universität Konstanz, Germany			
HEPES	(ICN Biomedicals, Ohio, USA)			

Table S2. Pressure wave data measured by different transducers at various positions.

Pressure wave data were measured at a distance of 3.5 cm from the nozzle by three different transducers at different positions: cracking pressure in the pressure reservoir (Rp), pressure peaks at thorax level with two transducers on the left and the right side (Pp(r), Pp(l)). The corresponding area under the curves (AUC(r), AUC(l)) and the duration of the pressure wave t(r), t(l) were calculated. Data are expressed as mean ± SD, the number of individual experiments (n), variation coefficient (CV).

	**Rp [bar]**	**Pp(r) [bar]**	**AUC(r) [V×s]**	**t(r) [µs]**	**Pp(l) [bar]**	**AUC(l) [V×s]**	**t(l) [µs]**
**mean±SD**	8.6 ± 0.2	2.7 ± 0.4	95. ± 6	612 ± 17	3.6 ± 0.9	101 ± 10	644. ± 55
**n**	218	219	220	219	220	218	217
**CV [%]**	2	14	6	3	26	10	9

Figures

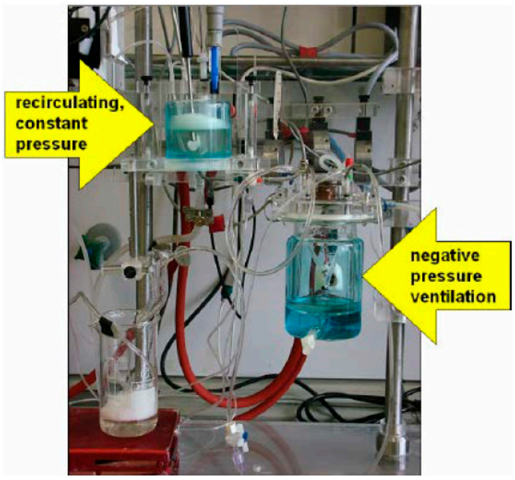

Figure S1. The Setup of the isolated perfused rat lung. The isolated rat lung is perfused in a recirculating fashion, blood-free under constant pressure conditions and with a negative pressure ventilation.

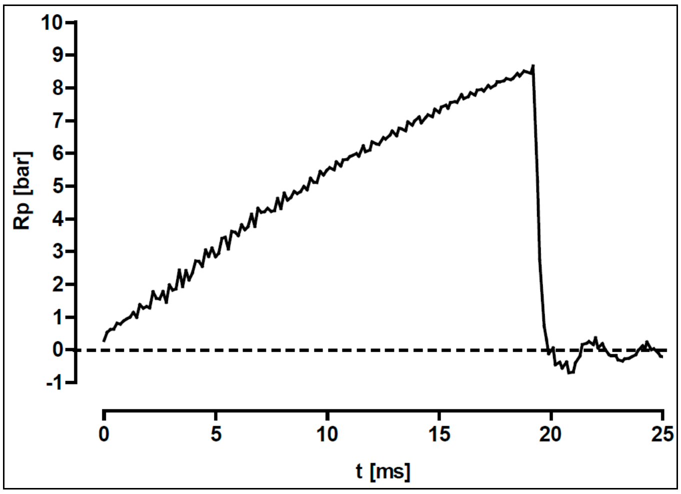

Figure S2. Time course of pressure increase in the pressure reservoir until rupture of the diaphragm. The working pressure in the compressed air bottle was 18 bar. Under control of a valve the pressure charged the pressure reservoir. The time course of the rupture pressure (Rp) was measured by the transducer in the reservoir.

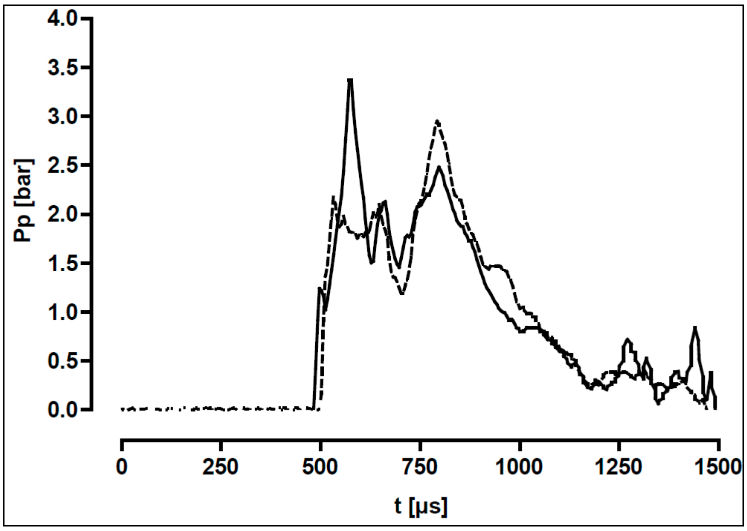

Figure S3. Pressure wave monitoring at rat thorax level. The pressure wave data were measured over time by two sensors on the right Pp(r) and left side Pp(l) of the rat. Pp(r): —,. n = 1; Pp(l): ---, n = 1.

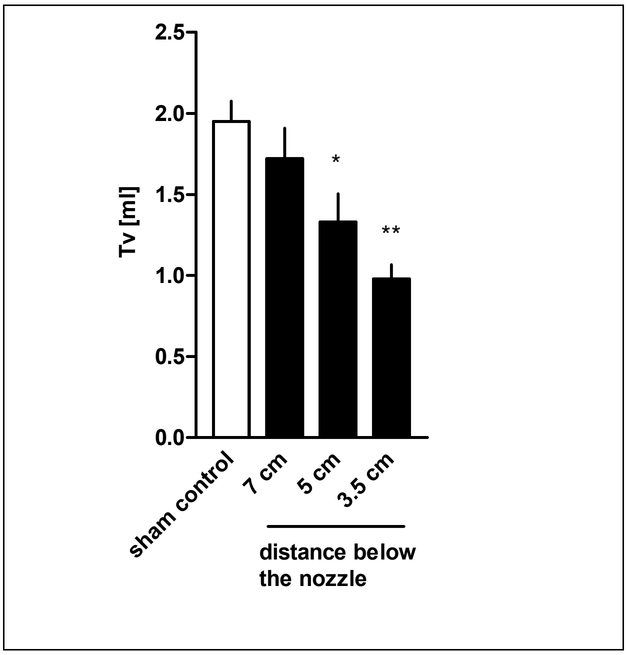

Figure S4. Pressure wave intensity-related decrease in initial tidal volume after thoracic trauma. Rats were exposed to the pressure wave at different distances from the nozzle (3.5 cm, n = 9; 5 cm, n = 16; 7 cm, n = 7). The rat lungs were perfused immediately after blast. Non-traumatized animals served as sham controls (n = 7). The initial tidal volume (Tv) was assessed with standard ventilation pressures (PEEP/ PIP: -2/ -7 cm H2O). Data are expressed as mean ± SD, number of experiments (n). Statistical analysis was performed by One-way ANOVA and Dunnett's Multiple Comparison Test: * $p < 0.05$ and ** $p < 0.01$ vs. sham control. Correlation between distance and Tv was performed by linear regression revealing a Pearson r of $r = 0.58$, $p = 0.0005$.
